# Letter to the Editor of the Journal of Medical Systems: Regarding “Responses of Five Different Artificial Intelligence Chatbots to the Top Searched Queries About Erectile Dysfunction: A Comparative Analysis”

**DOI:** 10.1007/s10916-024-02082-y

**Published:** 2024-07-05

**Authors:** Jakub Brzeziński, Robert Olszewski

**Affiliations:** National Institute of Geriatrics, Rheumatology and Rehabilitation, Spartańska 1 Street, Warsaw, 02-637 Poland

A recently published article in the journal, which is both intriguing and highly futuristic, examines the quality and readability of responses provided by five different AI-based chatbots on erectile dysfunction. In the ‘Materials and Methods’ section, the authors detail their analysis of these responses using various readability scales, including DISCERN, Ensuring Quality Information for Patients (EQIP), and the Flesch-Kincaid Grade Level (FKGL) and Reading Ease (FKRE). Additionally, they disclose the names of the five AI-based chatbots used to generate the responses under analysis [1].

Upon reviewing the article, we noticed an error in the chatbots mentioned by the authors. In the initial sentence of the abstract and the second paragraph of the ‘Materials and methods’ section, the authors introduced five chatbots: ChatGPT, Bard, Bing, Ernie, and Copilot. However, the authors used the same chatbots, Microsoft Bing and Copilot, throughout their article. They also included links to the chatbots’ websites in the second paragraph. We were directed to the same Copilot chatbot page upon visiting the provided Microsoft Bing: https://www.bing.com/chat and Copilot links: https://copilot.microsoft.com. This indicates a potential oversight on the part of the authors.

During Microsoft Ignite 2023, held from November 15–17, 2023, it was announced that the Bing chatbot would rebrand to Copilot, effective December 1, 2023. Copilot enhances the existing Bing service environment to deliver a novel search experience. Like Bing, Copilot is powered by a cutting-edge large language model (LLM) - GPT-4 (Generative Pre-trained Transformer-4). [2].

Contrary to the article’s statement, the authors did not use five different chatbots but four. Interestingly, one of these chatbots underwent a name change during the analysis. The authors failed to provide the specific dates when the chatbots were queried and analyzed, leaving the rationale for counting one chatbot as two unclear. Recent research articles on the utilization of chatbots powered by large language models (LLMs) have noted that Bing has been rebranded as Copilot [3].

I am contacting you not to undermine the authors’ work but to rectify any inaccuracies. This article might misinform readers interested in utilizing chatbots powered by large language models (LLMs). The authors propose using five distinct chatbots, when in reality, they employ four, as two are identical.

## Data Availability

All data supporting the findings of this study are available within the paper: Şahin MF, Ateş H, Keleş A, Özcan R, Doğan Ç, Akgül M, Yazıcı CM. Responses of Five Different Artificial Intelligence Chatbots to the Top Searched Queries About Erectile Dysfunction: A Comparative Analysis. J Med Syst. 2024 Apr 3;48(1):38. doi: 10.1007/s10916-024-02056-0. PMID: 38568432; PMCID: PMC10990980.
